# Supplementary material for: A conserved CCM complex promotes apoptosis non-autonomously by regulating zinc homeostasis
Source: Nat Commun. 2019 Apr 17;10:1791. doi: 10.1038/s41467-019-09829-z (PMC6470173; doi:10.1038/s41467-019-09829-z)
Supplement: Supplementary file 1 — Supplementary Information [file 41467_2019_9829_MOESM1_ESM.pdf]

Supplementary Information

A conserved CCM complex promotes apoptosis non-autonomously by regulating zinc homeostasis

Chapman *et al.*

Supplementary Table 1. List of experimental strains

Supplementary Table 2. List of antibodies

Supplementary Table 3. List of reagents

Supplementary Table 4. List of commercial assays

Supplementary Table 5. List of oligonucleotides

Supplementary Figure 1. KRI-1 regulates MPK-1/ERK1 to promote IR-induced apoptosis.

Supplementary Figure 2. *kri-1* suppressor screen identifies the ERK5/MAPK pathway and KLF-3.

Supplementary Figure 3. MPK-2 and KLF-3 regulate IR-induced apoptosis downstream of KRI-1.

Supplementary Figure 4. KRI-1 interacts with CCM-2, ICAP-1, and regulates transcription.

Supplementary Figure 5. Zinc transporters regulate IR-induced apoptosis.

Supplementary Figure 6. Zinc is a negative regulator of IR-induced apoptosis.

Supplementary Table 1. List of experimental strains

Strain	Source
N2 (Wild Type)	Caenorhabditis Genetics Center (CGC)
WD61 *kri-1(ok1251)*	Previously described[12]
SD551 *let-60(ga89)*	CGC
AH102 *lip-1(zh15)*	CGC
WD435 *kri-1(ok1251); lip-1(zh15)*	This study
WD338 *kri-1(ok1251); let-60(ga89)*	This study
WD439 *kri 1(ok1251); E02D9.1a(T503del)*	This study
WD440 *kri 1(ok1251); E02D9.1a(G717A)*	This study
WD441 *kri-1(ok1251); mpk-2(G741A)*	This study
WD442 *kri-1(ok1251); E02D9.1a(G213A)*	This study
WD443 *kri-1(ok1251); E02D9.1a(C541T)*	This study
WD444 *kri-1(ok1251); Y106G6A.1(G1179A)*	This study
WD445 *kri-1(ok1251); klf-3(G749T)*	This study
WD446 *kri-1(ok1251); Y106G6A.1(C1007T)*	This study
WD447 *kri-1(ok1251); Y106G6A.1(C1007T)*	This study
WD448 *kri-1(ok1251); Y106G6A.1(C1007T)*	This study
WD449 *kri-1(ok1251); E02D9.1a(G717A)*	This study
WD450 *kri-1(ok1251); Y106G6A.1(G949A)*	This study
WD451 *kri-1(ok1251); Y106G6A.1(C1007T)*	This study
CB1370 *daf-2(e1370)*	CGC
WD485 *mpk-2(ok219)* 2x outcrossed	This study
WD483 *kri-1(ok1251); mpk-2(ok219)*	This study
WD658 *klf-3(on34)* 2x outcrossed	This study
WD628 *kri-1(ok1251); klf-3(on34)*	This study
MT3970 *mab-5ced-9(n1653)*	CGC
WD682 N2 onEx93{*Pmpk-2::mpk-2;myo-2::rfp;myo-3::rfp*} #1	This study
WD683 N2 onEx94{*Pmpk-2::mpk-2;myo-2::rfp;myo-3::rfp*} #2	This study
WD684 N2 onEx95{*Pmpk-2::mpk-2;myo-2::rfp;myo-3::rfp*} #3	This study
WD685 N2 onEx96{*Pelt-2::mpk-2;myo-2::rfp;myo-3::rfp*} #1	This study
WD686 N2 onEx97{*Pelt-2::mpk-2;myo-2::rfp;myo-3::rfp*} #2	This study
WD687 N2 onEx98{*Pelt-2::mpk-2;myo-2::rfp;myo-3::rfp*} #3	This study
WD157 *kri-1(ok1251);* muEx353{*Pkri-1::gfp::kri-1;odr-1::rfp*}	Previously described[12]
VJ268 fgEx12{P*act-5*::*act-5*::*gfp*}	Previously described[33]
WD688 N2 onEx99 {*Pzipt-2.3::zipt-2.3::gfp; rol-6*} #1	This study
WD689 N2 onEx100 {*Pzipt-2.3::zipt-2.3::gfp; rol-6*} #2	This study
WD690 N2 onEx101 {*Pzipt-2.3::zipt-2.3::gfp; rol-6*} #3	This study
WD691 *kri-1(ok1251);* onEx99 {*Pzipt-2.3::zipt-2.3::gfp; rol-6*}	This study
WD675 *zipt-2.3(ok2094)* 4x outcrossed	This study
GH378 *pgp-2(kx48)*	CGC
N2 Ex{*Pmpk-2::gfp; rol-6*} #1	This study
N2 Ex{*Pmpk-2::gfp; rol-6*} #2	This study
N2 Ex{*Pmpk-2::gfp; rol-6*} #3	This study
RB2527 *ttm-1(ok3503)*	CGC
WU209 *cdf-1(n2527)*	CGC
Tg(kdrl:Hsa.HRAS-mcherry)^{s916,s896}	ZFIN
krit1^{ty219c}	Previously described[65]
Pdgfb-iCreERT2; *Krit1fl/fl*	Previously described[49]

Supplementary Table 2. List of antibodies

Antibody	Company	Catalogue Number
α-dpERK1 rabbit monoclonal	Cell Signaling	#4370
α-Nuclear Pore Complex mouse monoclonal (Mab414)	Abcam	#24609
goat α-rabbit Alexa 488	Invitrogen/Thermo Fisher	# A-11034
donkey α-mouse Alexa 568	Invitrogen/Thermo Fisher	# A10037
GFP-Trap_MA	ChromoTek	#gtma-10

Supplementary Table 3. List of reagents

Reagent	Company	Catalogue Number
EMS	Sigma	#M0880
Fluozin-3	Invitrogen/Thermo Fisher	#F24195
Zinpy-1	Cayman Chemical	# 288574-78-7
$ZnSO_4$	Sigma	#Z4750
Cas9	Integrated DNA Technologies	#1081060
Trizol	Invitrogen/Thermo Fisher	#15596026
PTU	Sigma	# 103-85-5
Ultra pure HNO_3	Fisher	#A509P500
30% H_20_2 for trace metal	Sigma	#7722-84-1
Zinc standard for ICP TraceCERT	Sigma	#18562-100ML-F
Mini Protease Inhibitor Cocktail	Sigma Millipore	# 11836153001
Phosphatase Inhibitor 2	Sigma	# P5726
Phosphatase Inhibitor 3	Sigma	# P0044
Trypsin	Millipore Sigma	# T6567

Supplementary Table 4. List of commercial assays

Assay	Company	Catalogue Number
DNeasy Blood & Tissue Kit	Qaigen	# 69504
Invitrogen First Strand Synthesis	Invitrogen/Thermo Fisher	#18080051
Random Hexamers	NEB	#S1330S
SYBR Green	BioRad	#1725271

Supplementary Table 5. List of oligonucleotides

Primer	Forward	Reverse
P*mpk-2* into pPD95.75	5'ttttttaccggtatgagtgcgagaactacgc3'	5'ttttttgggccccagaacccctgcaaccatc3'
mpk-2 into P*mpk-2*::pPD95.75	5'ttttttaccggtatgagtgcgagaactacgc3'	5'ttttttgggccccagaacccctgcaaccatc3'
mpk-2 into pJM559 (*Pelt-2*)	5'ttttttcggccgatgagtgcgagaactacgc3'	5'ttttttgggccccagaacccctgcaaccatc3'
P*zipt-2*.3::zipt-2.3 into pPD95.75	5'ttttttggatccggcatctaaactccctgaac3'	5'ttttttggtaccccggtagcccaaatcatgttgac3'
tbg-1 qPCR	5'cgtcatcagcctggtagaaca3'	5'tgatgactgtccacgttgga3'
zipt-2.3 qPCR	5'caccaacactcttcccttatt 3'	5' cccaggcttctaagcaatc3'
klf-3 crRNA guide:	5'gcucaugagcggacucacuc3'	
dpy-10 crRNA guide	5'gcuaccauaggcaccacgag3'	
klf-3(on34) repair oligo	5'tgcgaacttccaagaacagttttgccatttgcacacaaaaggtttctcaacagagtgagtccgctcatgagctttcaaatgtgaagacttagagtatcta3'	
dpy-10 repair oligo	5'cacttgaacttcaatacggcaagatgagaatgactggaaaccgtaccgcatgcggtgcctatggtagcggagcttcacatggcttcagaccaacagcctat3'	

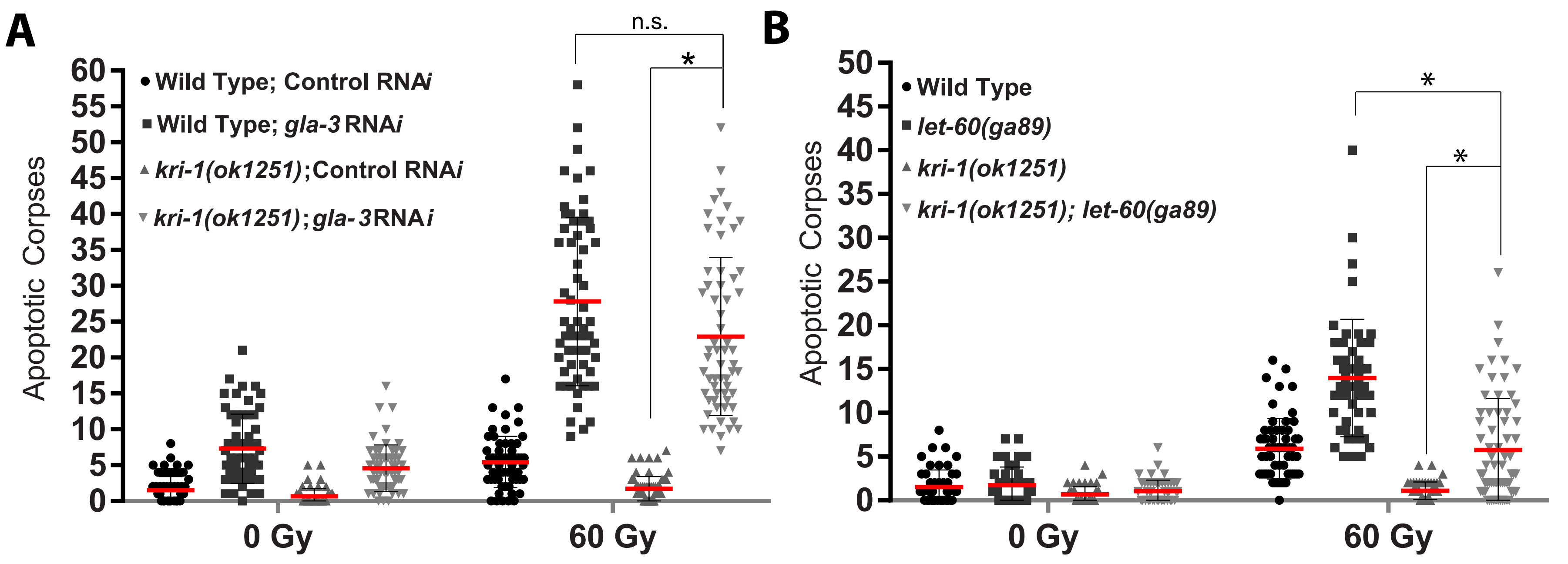

A
n.s.
*
Apoptotic Corpses
60
55
50
45
40
35
30
25
20
15
10
5
0
Wild Type; Control RNAi
Wild Type; gla-3 RNAi
kri-1(ok1251);Control RNAi
kri-1(ok1251);gla-3RNAi
0 Gy
60 Gy
B
*
*
Apoptotic Corpses
50
45
40
35
30
25
20
15
10
5
0
Wild Type
let-60(ga89)
kri-1(ok1251)
kri-1(ok1251); let-60(ga89)
0 Gy
60 Gy

Supplementary Figure 1. KRI-1 regulates MPK-1/ERK1 to promote IR-induced apoptosis. (A) IR-induced germline apoptosis scored after knock-down of *gla-3* in wild type, and *kri-1(ok1251)* animals (n≥60). (B) IR-induced germline apoptosis scored in wild type*, let-60(ga89), kri-1(ok1251)*, and *kri-1(ok1251); let-60(ga89)* animals (n≥60). (A-B) Red line is mean +/- standard deviation. Asterisk =P<0.05, two-sided, unpaired t-test.

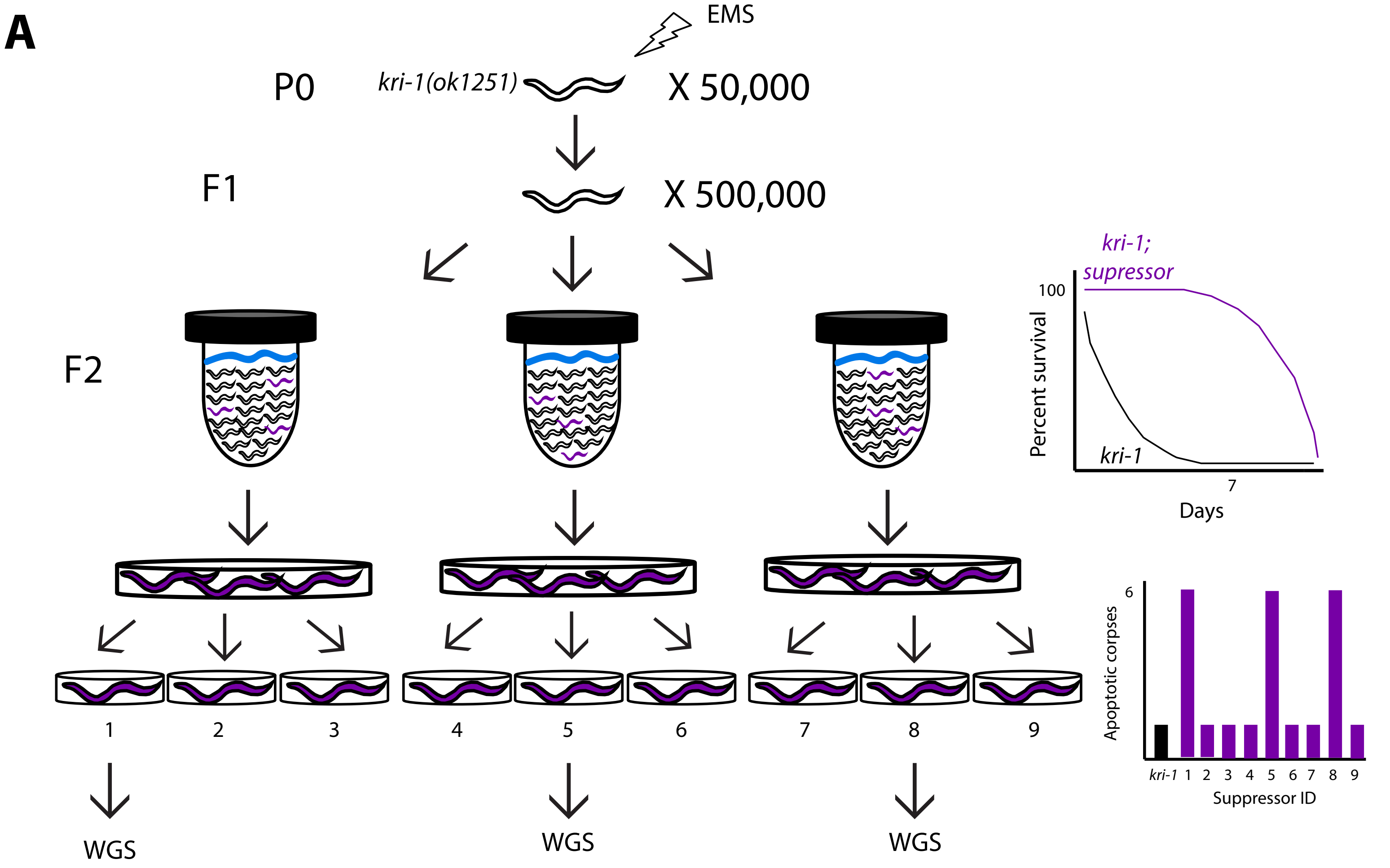

B

Strain Name	Mutated Gene	Human Homologue	Molecular Function	Chromosomal Location	Gene Region	Type of Variation	Type of Mutation
WD444	*Y106G6A.1*	MEKK3	MAP3K	ChrI: 9936362	Exon	Missense	Recessive
WD446	*Y106G6A.1*	MEKK3	MAP3K	ChrI: 9936582	Exon	Missense	Recessive
WD447	*Y106G6A.1*	MEKK3	MAP3K	ChrI: 9936582	Exon	Missense	Recessive
WD448	*Y106G6A.1*	MEKK3	MAP3K	ChrI: 9936582	Exon	Missense	Recessive
WD450	*Y106G6A.1*	MEKK3	MAP3K	ChrI: 9937149	Exon	Missense	Recessive
WD451	*Y106G6A.1*	MEKK3	MAP3K	ChrI: 9936582	Exon	Missense	Recessive
WD439	*E02D9.1*	MEK5	MAP2K	ChrI: 6802927	Exon	Frameshift Deletion	Recessive
WD440	*E02D9.1*	MEK5	MAP2K	ChrI: 6803701	Exon	Missense	Recessive
WD442	*E02D9.1*	MEK5	MAP2K	ChrI: 6802412	Intron	Splicing	Recessive
WD443	*E02D9.1*	MEK5	MAP2K	ChrI: 6803099	Exon	Missense	Recessive
WD449	*E02D9.1*	MEK5	MAP2K	ChrI: 6803990	Exon	Missense	Recessive
WD441	*mpk-2*	ERK5	MAPK	ChrII: 5106085	Intron	Splicing	Recessive
WD445	*klf-3*	Group 2 KLF (KLF1/2/4/5/6/7)	Transcription Factor	ChrII: 6621660	Exon	Missense	Dominant

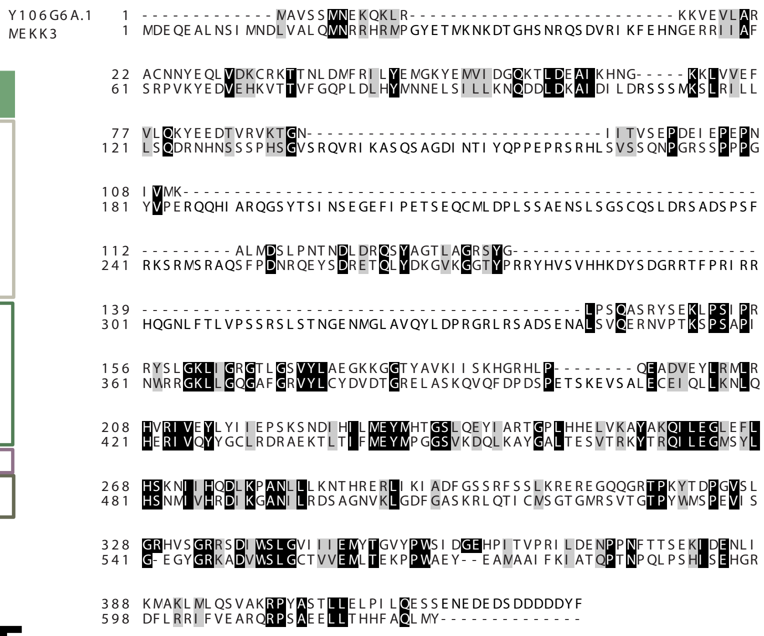

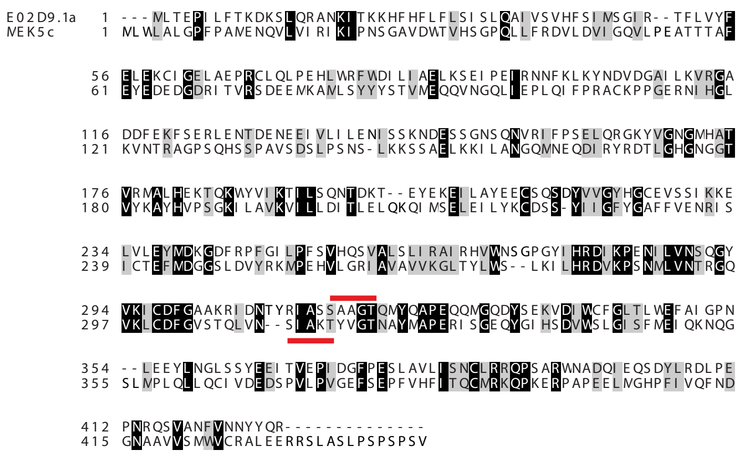

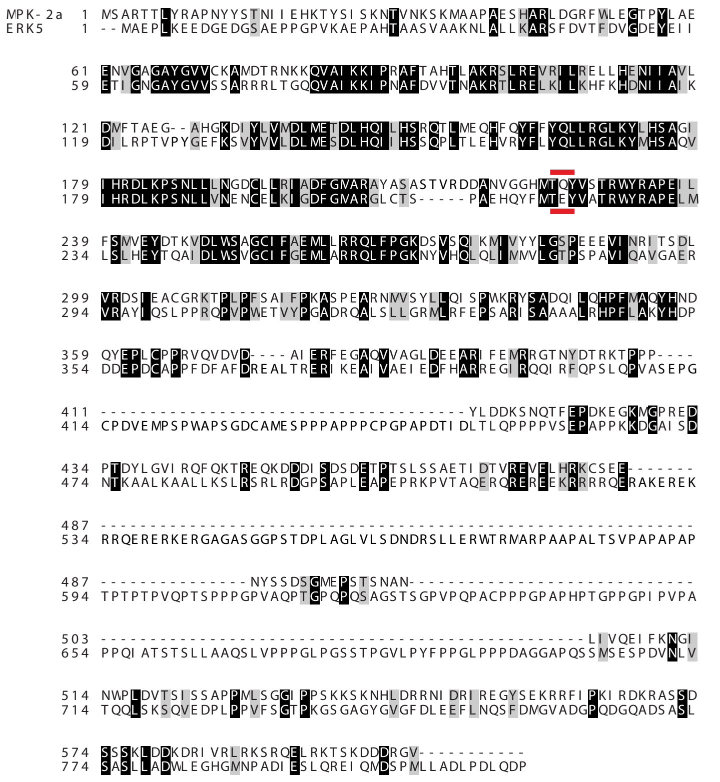

Supplementary Figure 2. *kri-1* suppressor screen identifies the ERK5/MAPK pathway and KLF-3. (A) Schematic of the *kri-1(ok1251)* suppressor screen. (B) *kri-1(ok1251)* suppressor candidates with restored apoptosis. (C-E). Alignment of Y106G6A.1, E02D9.1 (isoform a), and MPK-2 (isoform a) with human MEKK3 (isoform 1), MEK5 (isoform c), and ERK5 (isoform 1), respectively using ClustalW. Scores are 20.5 (C), 16.9 (D), and 29.6 (E) and represent the number of identities divided by the length of the alignment, as a percent. Black background represents identical amino acids and grey background indicates strong similarity. The Red line in (D) encompasses the conserved MEKK3 phosphorylation sites on MEK5 (Ser311, Thr315) and the red line in (E) encompasses the conserved MEK5 phosphorylation site on ERK5 (Thr218, Tyr220).

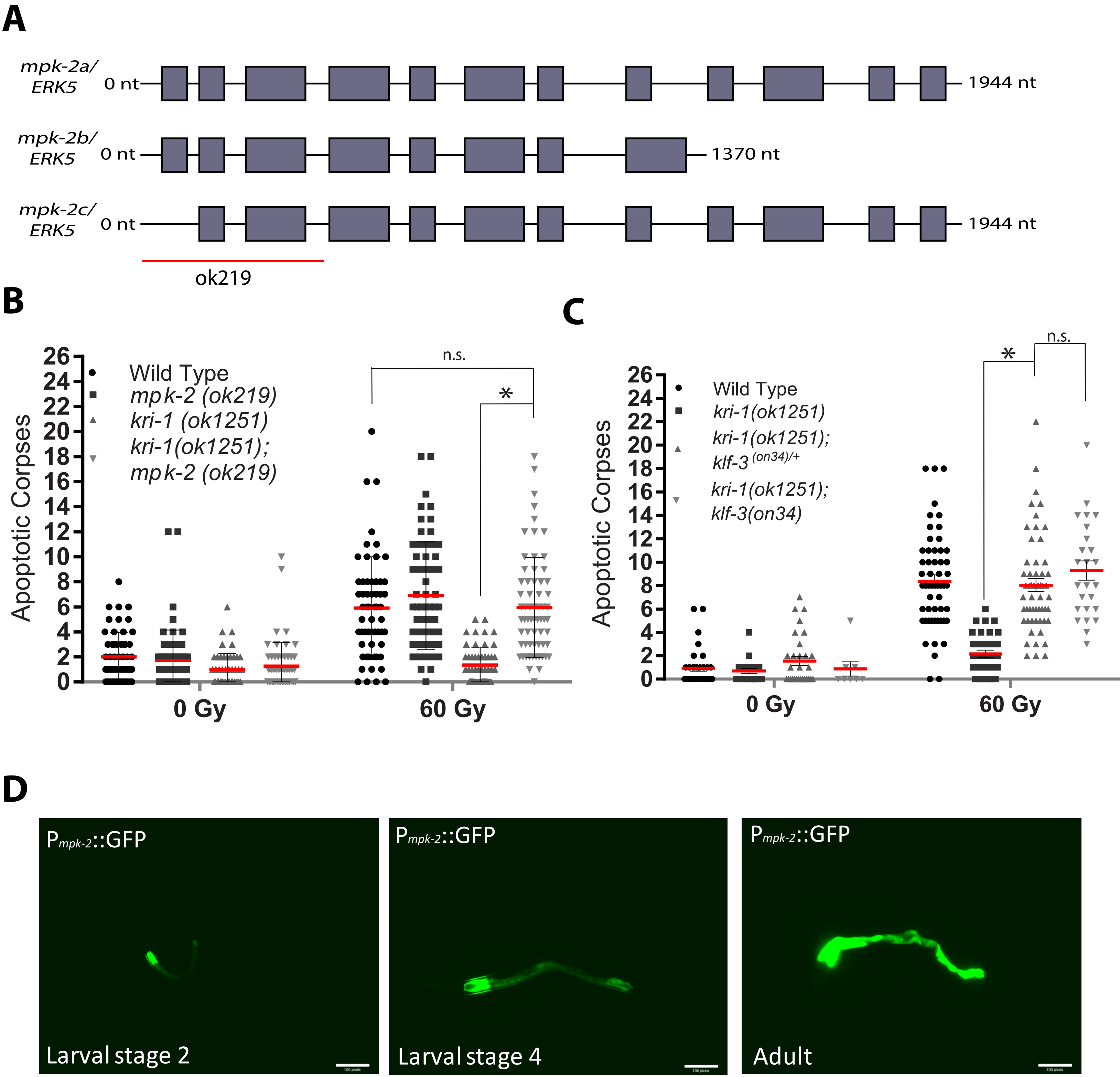
A
mpk-2a/ ERK5
0 nt
1944 nt
mpk-2b/ ERK5
0 nt
1370 nt
mpk-2c/ ERK5
0 nt
1944 nt
ok219
B
Apoptotic Corpses
Wild Type
mpk-2 (ok219)
kri-1 (ok1251)
kri-1(ok1251); mpk-2 (ok219)
n.s.
*
0 Gy
60 Gy
C
Apoptotic Corpses
Wild Type
kri-1(ok1251)
kri-1(ok1251); klf-3 (on34)/+
kri-1(ok1251); klf-3(on34)
*
n.s.
0 Gy
60 Gy
D
Pmpk-2::GFP
Larval stage 2
Pmpk-2::GFP
Larval stage 4
Pmpk-2::GFP
Adult

Supplementary Figure 3. MPK-2 and KLF-3 regulate IR-induced apoptosis downstream of KRI-1. (A) The *mpk-2(ok219)* allele consists of a 985 base pair out-of-frame deletion, removing the 5' UTR and the first three exons of isoform a and b, and the first two exons of isoform c. (B) IR-induced germline apoptosis scored in wild type, *mpk-2(ok219), kri-1(ok1251),*and *kri-1(ok1251); mpk-2(ok219)* animals (n≥50). (C) IR-induced germline apoptosis scored in wild type, *kri-1(ok1251), kri-1(ok1251); klf-3*$^{(on34)/+}$ and *kri-1(ok1251); klf-3(on34)* animals (n≥30). (D) Expression of $P_{mpk\text{-}2}$::*gfp* throughout development. Images are representative of three independent lines. Scale bar is 50 μm. (B-C) Red line is mean +/- standard deviation. Asterisk =P<0.05, two-sided, unpaired t-test.

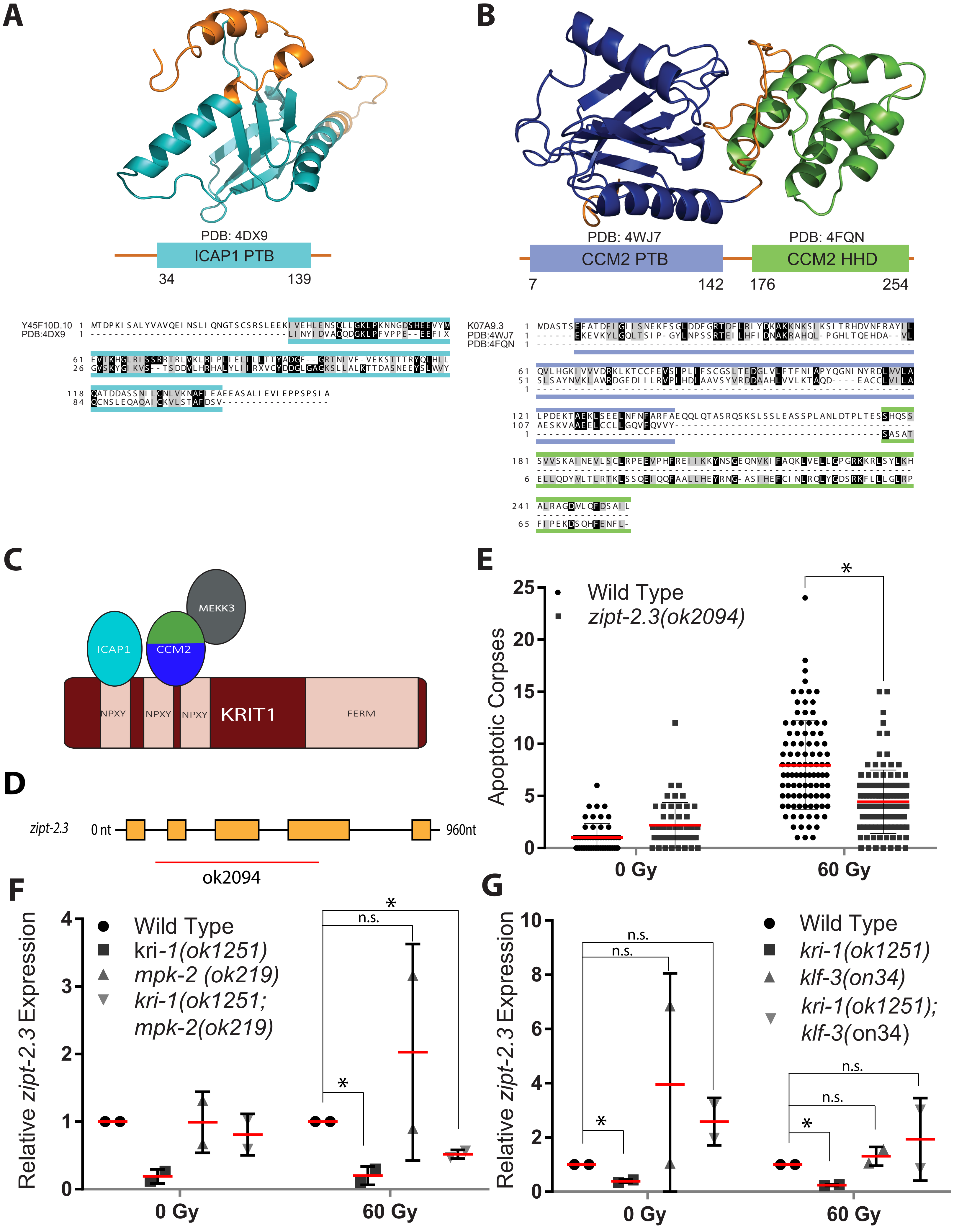

A
B
PDB: 4DX9
ICAP1 PTB
34
139
PDB: 4WJ7
PDB: 4FQN
CCM2 PTB
CCM2 HHD
7
142
176
254
C
MEKK3
ICAP1
CCM2
NPXY
NPXY
NPXY
KRIT1
FERM
D
zipt-2.3
0 nt
960nt
ok2094
E
Wild Type
zipt-2.3(ok2094)
Apoptotic Corpses
0 Gy
60 Gy
F
Relative zipt-2.3 Expression
Wild Type
kri-1(ok1251)
mpk-2 (ok219)
kri-1(ok1251; mpk-2(ok219)
n.s.
0 Gy
60 Gy
G
Relative zipt-2.3 Expression
Wild Type
kri-1(ok1251)
klf-3(on34)
kri-1(ok1251); klf-3(on34)
n.s.
0 Gy
60 Gy

Supplementary Figure 4. KRI-1 interacts with CCM-2, ICAP-1, and regulates transcription. (A) Alignment of Y45F10D.10 and human ICAP1. (B) Alignment of K07A9.3 and human CCM2. (A-B) Black background represents identical amino acids, and grey background indicates strong similarity. (C) Schematic of ICAP1 and CCM2 interacting with mammalian KRIT1. (D) The *zipt-2.3(ok2094)* allele consists of a 1561bp deletion which removes the second, third, and half of the fourth exon, resulting in an out-of-frame sequence. (E) IR-induced germline apoptosis scored in wild type, and *zipt-2.3(ok2094)* animals ($n \geq 50$). (F-G) Relative fold expression of *zipt-2.3* in *kri-1(ok1251), mpk-2(ok219)*, and *kri-1(ok1251); mpk-2(ok219)* animals (F) or *kri-1(ok1251), klf-3(on34)*, and *kri-1(ok1251); klf-3(on34)* animals (G) compared to wild type. Graphs (F &G) represent two biological replicates. (E-G) Red line is mean +/- standard deviation. Asterisk =$P<0.05$, two-sided, unpaired t-test.

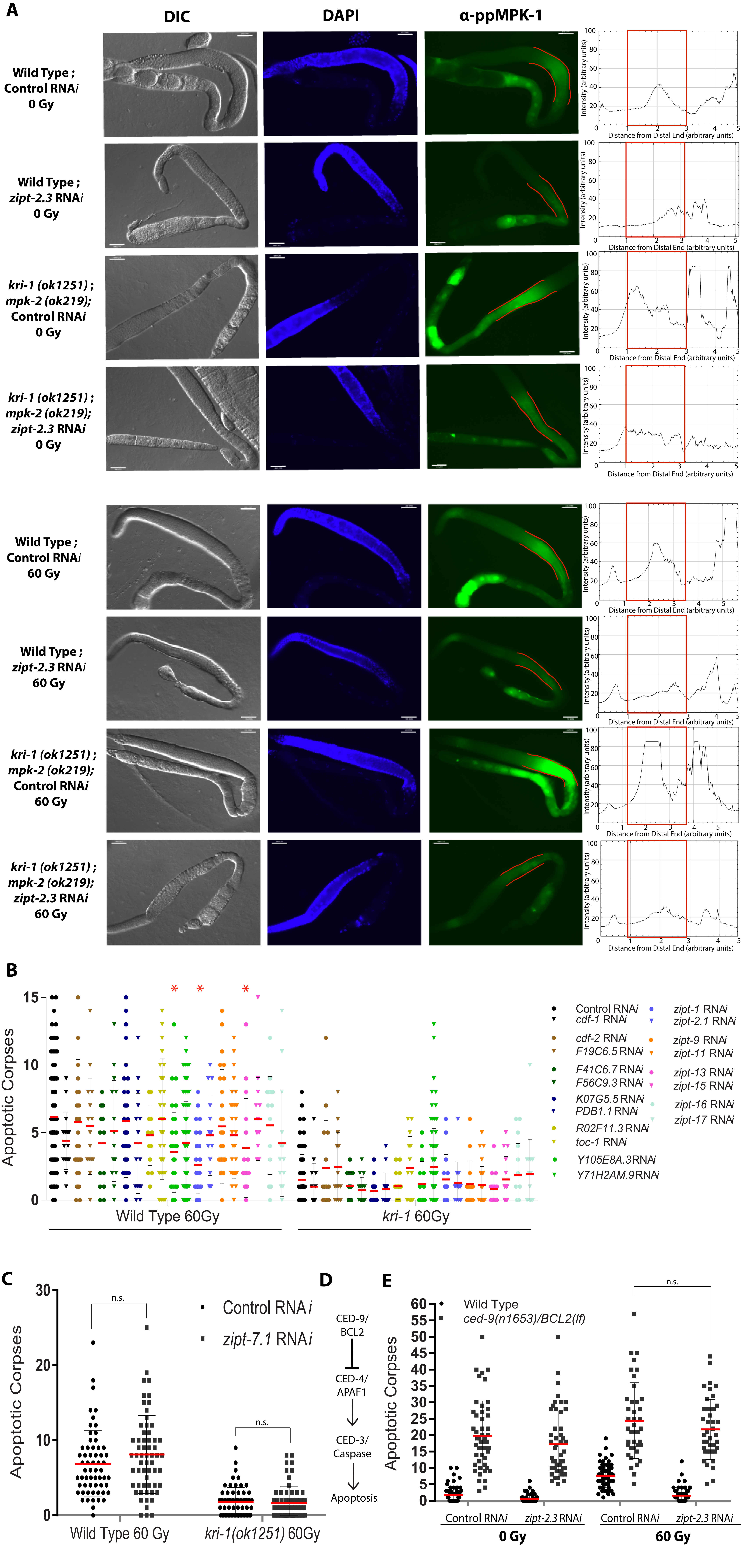
A
DIC
DAPI
α-ppMPK-1
Wild Type ; Control RNAi 0 Gy
Wild Type ; zipt-2.3 RNAi 0 Gy
kri-1 (ok1251) ; mpk-2 (ok219); Control RNAi 0 Gy
kri-1 (ok1251) ; mpk-2 (ok219); zipt-2.3 RNAi 0 Gy
Wild Type ; Control RNAi 60 Gy
Wild Type ; zipt-2.3 RNAi 60 Gy
kri-1 (ok1251) ; mpk-2 (ok219); Control RNAi 60 Gy
kri-1 (ok1251) ; mpk-2 (ok219); zipt-2.3 RNAi 60 Gy
Intensity (arbitrary units)
Distance from Distal End (arbitrary units)
B
Apoptotic Corpses
Wild Type 60Gy
kri-1 60Gy
Control RNAi
cdf-1 RNAi
cdf-2 RNAi
F19C6.5 RNAi
F41C6.7 RNAi
F56C9.3 RNAi
K07G5.5 RNAi
PDB1.1 RNAi
R02F11.3 RNAi
toc-1 RNAi
Y105E8A.3RNAi
Y71H2AM.9RNAi
zipt-1 RNAi
zipt-2.1 RNAi
zipt-9 RNAi
zipt-11 RNAi
zipt-13 RNAi
zipt-15 RNAi
zipt-16 RNAi
zipt-17 RNAi
C
Apoptotic Corpses
n.s.
Control RNAi
zipt-7.1 RNAi
Wild Type 60 Gy
kri-1(ok1251) 60Gy
D
CED-9/ BCL2
CED-4/ APAF1
CED-3/ Caspase
Apoptosis
E
Apoptotic Corpses
Wild Type
ced-9(n1653)/BCL2(lf)
n.s.
Control RNAi
zipt-2.3 RNAi
0 Gy
60 Gy

Supplementary Figure 5. Zinc transporters regulate IR-induced apoptosis. (A) di-phosphorylated MPK-1 in the germlines of wild type; control RNA*i*, wild type; *zipt-2.3* RNA*i*, *kri-1(ok1251); mpk-2(ok219)*; control RNA*i*, and *kri-1(ok1251); mpk-2(ok219)*; *zipt-2.3* RNA*i* animals is measured along the midline from the distal to proximal end. The red box on each graph corresponds to the pachytene region. Images are representative of two independent experiments and at least 20 worms per strain, per condition (400x magnification). Scale bar is 25 μm. (B) IR-induced germline apoptosis scored in wild type and *kri-1(ok1251)* worms after knock-down of 19 zinc transporter genes. Red asterisk indicates significance compared to control. The graph represents at least one independent replicate (n≥15). (C) IR-induced germline apoptosis scored in wild type and *kri-1(ok1251)* worms after knock-down of *zipt-7.1* (n≥50). (D) Schematic of the core apoptosis cascade. (E) IR-induced germline apoptosis scored in wild type and *ced-9(n1653)* animals after knock-down of *zipt-2.3* (n≥40). (B-E) Red line is mean +/- standard deviation. Asterisk =P<0.05, two-sided, unpaired t-test.

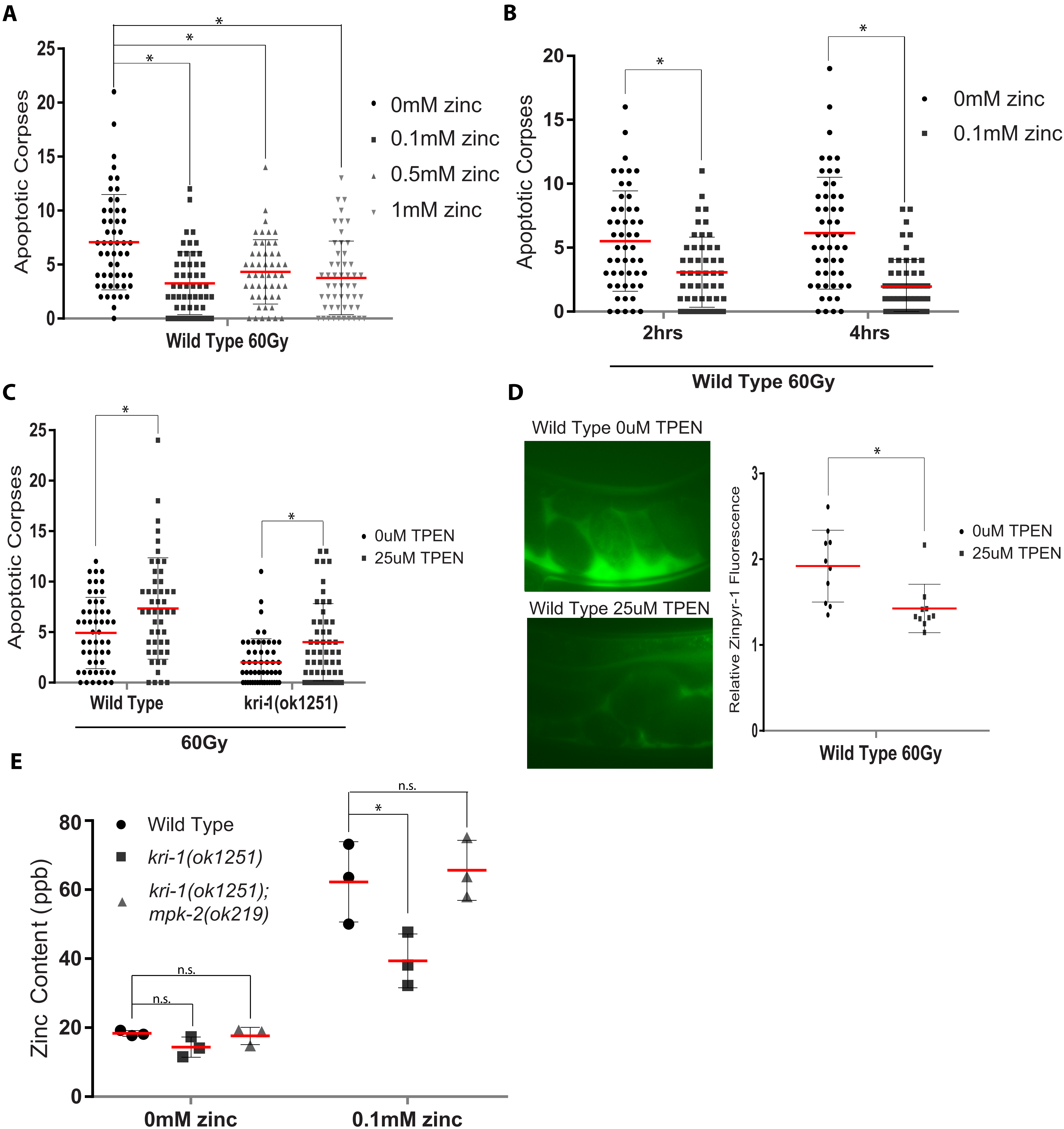
A
Apoptotic Corpses
0mM zinc
0.1mM zinc
0.5mM zinc
1mM zinc
Wild Type 60Gy
B
Apoptotic Corpses
0mM zinc
0.1mM zinc
2hrs
4hrs
Wild Type 60Gy
C
Apoptotic Corpses
0uM TPEN
25uM TPEN
Wild Type
kri-1(ok1251)
60Gy
D
Wild Type 0uM TPEN
Wild Type 25uM TPEN
Relative Zinpyr-1 Fluorescence
0uM TPEN
25uM TPEN
Wild Type 60Gy
E
Zinc Content (ppb)
Wild Type
kri-1(ok1251)
kri-1(ok1251); mpk-2(ok219)
n.s.
0mM zinc
0.1mM zinc

Supplementary Figure 6. Zinc is a negative regulator of IR-induced apoptosis. (A) IR-induced germline apoptosis scored in wild type animals after exposure to growth media with 0mM, 0.1mM, 0.5mM, and 1mM zinc (n≥50). (B) IR-induced germline apoptosis scored in wild type animals after exposure to liquid buffer with 0mM and 0.1mM zinc (n≥50). C) IR-induced germline apoptosis scored in wild type and *kri-1(ok1251)* worms after exposure to liquid buffer with 0μM and 25μM TPEN (n≥50). (D) Relative Zinpyr-1 fluorescence in the body cavity of wild type animals after incubation with 0μM and 25μM TPEN (n≥10). (E) Zinc measured by ICP-MS in wild type, *kri-1(ok1251),* and *kri-1(ok1251); mpk-2(ok219)* animals after exposure to 0mM and 0.1mM zinc (n≥15,000). (A-E) Red line is mean +/- standard deviation. Asterisk =P<0.05, two-sided, unpaired t-test.
